# Improving Surveillance and Epidemic Response in Ohio Childcare Settings

**DOI:** 10.3390/ijerph192416927

**Published:** 2022-12-16

**Authors:** Darcy A. Freedman, Timothy H. Ciesielski, Owusua Yamoah, Elaine A. Borawski, Kristie R. Ross, Nora L. Nock, Eun Kyung Lee, Anastasia Dimitropoulos, Sonia Minnes, Kimberly Burkhart, Callie Ogland-Hand, Daniel J. Tisch

**Affiliations:** 1 Mary Ann Swetland Center for Environmental Health, School of Medicine, Case Western Reserve University, Cleveland, OH 44106, USA; 2 Department of Population and Quantitative Health Sciences, School of Medicine, Case Western Reserve University, Cleveland, OH 44106, USA; 3 Schubert Center for Child Studies, Case Western Reserve University, Cleveland, OH 44106, USA; 4 Department of Nutrition, School of Medicine, Case Western Reserve University, Cleveland, OH 44106, USA; 5 University Hospitals of Cleveland, Cleveland, OH 44106, USA; 6 Department of Health Law, School of Public Health, Boston University, Policy and Management, Boston, MA 02118, USA

**Keywords:** COVID-19, childcare, infectious disease control, occupational health

## Abstract

At the start of the Coronavirus Disease of 2019 (COVID-19) pandemic, the risk of cases in childcare programs was unknown. Thus, a rapid-response research approach was launched in Ohio childcare settings. Passive surveillance data from a state-operated incident reporting system were evaluated to estimate the number of COVID-19 cases from 15 August 2020 to 1 January 2021. Additionally, active surveillance with self-administered reverse transcriptase–polymerase chain reaction (RT-PCR) tests were conducted among staff at 46 childcare programs. Finally, six zoom-based focus groups with program administrators were used to gain feedback. Staff and children in childcare settings contributed 0.38% and 0.15% of the COVID-19 cases in Ohio during this timeframe, respectively. RT-PCR testing identified 3 unrecognized cases (0.88% of tests), and all occurred when the statewide positivity rate was >5%. Focus groups revealed that access to affordable cleaning supplies, masks, and reliable staffing were critical. Perhaps most importantly, we conclude that expanding the incident reporting system to include a childcare census would allow for the tracking of future health problems with highly valuable incidence rate estimations.

## 1. Introduction

Epidemiologic studies conducted at the start of the pandemic (March–June 2020) found low rates of severe acute respiratory syndrome coronavirus 2 (SARS-CoV-2) transmission and high rates of mitigation compliance in U.S. childcare settings [1]. However, they also documented asymptomatic transmission events [2] and revealed the deep impact of childcare closures on workforce engagement among parents [3]. Overall, this evidence indicated that the Centers for Disease Control and Prevention and state guidelines for reducing transmission in childcare facilities were effective when community transmission rates were low. However, it was not clear if transmission among children and staff in childcare would increase when community transmission increased. To address this gap, the Ohio Bureau of Workers’ Compensation reached out to the academic research community in late spring of 2020 to conduct a rapid assessment of the states’ childcare programs. In response, we implemented a study to (1) conduct surveillance for COVID-19 cases within Ohio childcare programs during the fall of 2020 and (2) assess what was needed to maintain the safety of childcare settings. While vaccines are now available for those over 6 months of age, we still have much to learn about protecting people in childcare settings during this pandemic and the next. Thus, our findings offer key insights into the surveillance and non-pharmacologic mitigation strategies needed to protect the workplace that enable all other workplaces.

## 2. Materials and Methods

### 2.1. Passive Statewide Surveillance Data

We evaluated passive statewide surveillance data to determine the weekly number of COVID-19 cases within childcare settings from 15 August 2020 to 1 January 2021. Information on COVID-19 cases among childcare workers and children was obtained from the Ohio Department of Job and Family Services (ODJFS) Serious Incident/Injury Reporting System. This surveillance system captured worksite-reported cases of COVID-19 at all licensed Ohio childcare programs. These reports are mandated, and failure to record an incident, such as a COVID-19 case, lowers a program’s quality rating [4]. ODJFS provided the research team with cumulative case numbers by county on a weekly basis for 20 weeks, and weekly case numbers were calculated from these data. Statewide COVID-19 case numbers for this study period were downloaded on 19 January 2021 from the Ohio Department of Health COVID-19 Dashboard to provide a community reference [5,6]. SARS-CoV-2 test positivity rates were defined as the 7-day moving average from the last day of each week [5,6].

### 2.2. Active Surveillance in Select Ohio Counties

Active surveillance occurred at childcare programs located within 10 counties that collectively contain 64% of all licensed childcare programs in Ohio. These counties were selected to achieve variability in geographic location, population density, sociodemographic characteristics, and COVID-19 burden as of early June 2020. Program administrators from licensed sites in these counties were invited to join the study in July 2020, where eligible sites had at least five people total (staff and children), were open by August 2020, and were able to email study information to staff and families to conduct physically distanced study activities. Forty-six programs were enrolled, including 40 childcare centers and 6 family home providers. Within these programs, participants completed an online enrollment survey and then had the option to participate in (1) weekly online surveys documenting COVID-19-related symptoms they were experiencing, (2) self-administered RT-PCR lower nasal swab SARS-CoV-2 testing, and (3) focus groups. Entry into the weekly symptom surveys (n = 208) and SARS-CoV-2 testing (n = 167) was self-selected and open to all staff. The six focus groups were self-selected and open only to childcare program administrators (n = 28). Informed consent was provided separately and electronically for each data collection method. The study was approved by the Case Western Reserve University Institutional Review Board (STUDY20200949) and conformed with recognized ethical standards for research with human subjects.

The one-time online enrollment survey included self-reports of demographics, family structure, and health status. To identify unrecognized SARS-CoV-2 cases among childcare staff, RT-PCR testing of lower nasal samples was used (Food and Drug Administration emergency use-approved collection kit, Lets-Get-Checked, New York, NY, USA). The swabs were self-administered and mailed in for analysis. All enrolled childcare workers were eligible to complete testing up to three times from September to December 2020. The tests were mailed out in batches from September through November. The testing strategy provided multiple cross-sectional assessments of currently working staff. The participants received USD 50 for completing their first two tests. Brief surveys assessed symptoms experienced over the past week. Participants completing the brief survey were entered into a weekly raffle for a USD 10 gift card, and 10 cards raffled per week.

### 2.3. Focus Groups

Among the workers enrolled in the study, those identifying as administrators were invited to participate in one focus group conducted via an electronic video conferencing platform. In total, six focus groups were conducted. Each lasted 90 min, and the participants (n = 28) were compensated with USD 50 for their time. The focus groups included open-ended questions about opportunities and barriers to reducing the spread of COVID-19 in childcare programs. The sessions were recorded and transcribed verbatim. These data were analyzed using deductive coding based on the focus group guide as well as inductive coding of emergent themes. Coding by four team members (O.Y., C.O.-H., D.A.F. and E.A.B.) was conducted using NVivo Plus v12 (QSR International Pty Ltd., Burlington, MA, USA).

### 2.4. Quantitative Analyses

Wilcoxon–Mann–Whitney tests were used to assess if the number of childcare cases or their percentage among statewide cases were significantly different in the weeks with high versus low community transmission (threshold: 5% positivity rate [7]). Analyses were conducted with SAS 9.4 (Cary, NC, USA). Estimates for the total number of staff and children in attendance at Ohio childcare programs were not available, precluding calculation of the incidence rates or cumulative incidence within these groups.

## 3. Results

Evaluation of statewide COVID-19 data among childcare programs in Ohio during the last 20 weeks of 2020 revealed 2361 cases among workers and 914 among children. During this same timeframe, there were 618,430 total cases in Ohio, with workers and children in childcare settings contributing 0.38% and 0.15% of the statewide cases, respectively. Community transmission was low in the first 10 weeks of this study (≤5% SARS-CoV-2 test positivity rate [7]) and high (>5%) in the second 10 weeks of observation (Table 1). New COVID-19 cases per week among childcare workers (*p* < 0.001) and children (*p* < 0.001) were significantly higher in the weeks with high community transmission. However, the weekly percentage of statewide cases that were from childcare workers (*p* = 0.52) and children (*p* = 0.42) did not significantly change during the 10 weeks of high community transmission [8,9,10].

Within the 10 selected counties, 228 childcare workers enrolled in the study, representing about 40% of the eligible staff at the enrolled sites based on the self-reporting of childcare administrators. Most participants were female (96.1%) with a mean age of 42.1 years. The racial demographics were representative of the state, with 79.8% identifying as white and 14.5% as black. Most of the workers were classroom teachers (50.4%), administrators (26.3%), or teachers’ aides (10.1%) working with children of all ages, including infants (19.7%), toddlers (22.1%), preschoolers (36.3%), and school-aged children (12.1%).

In the active surveillance of adult childcare workers, 358 RT-PCR tests were completed, and 341 (95.3%) of these yielded definitive results (Table 2). Indeterminant results were due to user error related to sample collection or registration. Three positive tests were identified, resulting in a 0.88% test positivity rate among workers who volunteered to be tested. The positive cases occurred during the period of high community transmission (statewide positivity rate > 5%). Although this screening was designed to detect SARS-CoV-2 in asymptomatic individuals, the three positive cases each reported a mild nonspecific symptom during the week they tested positive. One participant reported a headache, and two reported congestion or a runny nose.

Six focus groups with 28 childcare administrators revealed that childcare programs were investing substantial effort to comply with statewide mitigation guidelines to reduce the transmission of COVID-19 (Table 3). Several challenges were identified that limited worksite capacity to fully and sustainably comply with guidelines and reduce SARS-CoV-2 transmission in childcare settings. First, there was consensus that operating childcare programming at lower teacher-to-student ratios to promote physical distancing was costly and unsustainable. A variety of specific new costs contributed to this strain, including the purchase of additional learning resources to limit the number of children using common supplies and the provision of Wi-Fi to support remote learning for school-aged children. Second, maintaining staffing capacity amid a global pandemic was identified as a significant challenge. Additional workers were needed for symptom screening, managing outside child drop-off and pick-up procedures, cleaning and sanitizing multiple times per day, and managing school-aged children who were engaged in remote learning. While workers were encouraged to stay home if sick, lack of access to temporary workers could result in the closure of a classroom. Third, administrators cited limited access to cost-efficient procurement systems to buy personal protective equipment, cleaning supplies, and COVID-19 tests (i.e., tests for use beyond this study). Some of them reported shopping during early hours to secure masks, gloves, and cleaning supplies such as wipes, bleach, and bottles of alcohol. Others shared that they made their own cleaning supplies due to supply chain barriers. The participants also noted that prices for personal protective equipment and cleaning materials had increased significantly.

Key Messages: Rapid outreach and funding offers from a state agency at the start of the pandemic attracted academics to quickly implement childcare research that the state was not staffed to conduct. The preexistence of a license-mandated incident reporting system allowed researchers to track the number of COVID-19 cases in Ohio childcare settings. Highly valuable incidence rates could have been obtained for decision making if the numbers of staff and children in Ohio childcare programs were also routinely tracked. In late 2020, childcare-related COVID-19 cases represented a small percentage of the total cases in Ohio, with most cases occurring when statewide positivity rates were over 5%. The focus groups revealed that childcare programs need affordable supplies, personal protective equipment, and reliable staffing to continue non-pharmacologic mitigation strategies in settings with young unvaccinated children.

## 4. Discussion

Passive surveillance with state agency data revealed that workers and children in childcare made up a small yet consistent percentage of the COVID-19 cases in Ohio during the last 20 weeks of 2020 (0.38% and 0.15%, respectively), and these percentages did not significantly differ in the 10 weeks when transmission rates were high (>5% positivity rate). If the statewide incident system had been equipped to collect the numbers of staff and children in Ohio childcare settings, then valuable incidence rates could have been calculated for government decision making. Perhaps most importantly, the incidence rates would allow for relative risk comparisons with other work and school settings. There will always be tradeoffs associated with opening or closing childcare programs, schools, and workplaces [8,9,10,11,12,13,14], but we cannot effectively assess these tradeoffs if we cannot quickly estimate transmission risk.

Active “asymptomatic” surveillance in 10 counties revealed a small percentage (0.88%) of staff volunteers who tested positive for SARS-CoV-2. The positive tests occurred during the period of high community transmission, and each person had at least one mild, nonspecific symptom. Overall, active surveillance with self-administered SARS-CoV-2 tests proved useful for detecting unrecognized cases among childcare workers, and this screening may have its greatest utility when community transmission rates are high.

The focus group findings illuminated the barriers to COVID-19 mitigation in childcare settings, including reduced financial capacity to operate childcare programming with fewer students, limited access to reliable childcare staffing, and a lack of access to cost-efficient supplies (personal protective equipment, cleaning supplies, and COVID-19 tests). Many of these barriers are still in place.

In terms of limitations, the active surveillance diagnostic tests were self-administered, and the data for passive surveillance came from self-reporting to ODJFS. However, the potential for underreporting to ODJFS was discouraged through the state’s licensing system, which penalizes a program’s quality rating if an unreported COVID-19 case is identified. We do not have information on how many programs received these penalties, and this hampered our ability to estimate the potential error in ODJFS case numbers. Estimates for the total number of staff and children in attendance at Ohio childcare programs were not available, so we were unable to calculate incidence rates or cumulative incidence within these groups. However, this limitation represents a key insight for enhancing the utility of the incident reporting system in epidemics: attendance data provide critical context and should be routinely collected. We also note that statewide surveillance was aggregated at the county level, and this prevented examination of COVID-19 cases by childcare sites. Finally, we note that the focus group findings may not generalize to all childcare worksites.

These findings align with US studies [1,2,3] and European evaluations [15,16,17] which indicated that childcare settings did not present elevated risk to the staff, children, or community when SARS-CoV-2 transmission mitigation strategies were in place. However, they also revealed that COVID-19 cases were more likely in childcare settings when community positivity rates were high. This is somewhat reassuring, but we note that new variants continue to change the situation, and the need for improved surveillance strategies is clear. Every childcare-related case has both health and economic consequences related to the role of childcare in supporting workforce participation (especially for women). [18] Overall, our findings provide a childcare research model for epidemic response, and they highlight the utility of collecting attendance information. Importantly, our findings also highlight what is needed to sustain non-pharmacological mitigation strategies. These three insights may be critical in our responses to future pandemics.

## 5. Conclusions

All public health surveillance strategies have strengths and weaknesses, but the preexistence of a license-mandated incident reporting system for childcare worksites in Ohio provided critical pandemic-related data. Furthermore, rapid-response collaborations between state agencies and academic research institutions provided infrastructure for active surveillance to corroborate and extend the information from passive state-mandated reporting. In this collaboration, we also learned that the routine collection of attendance data could vastly expand the utility of the state’s incident reporting data. This would be helpful in infectious epidemic responses, but it would also aid in the tracking of incidence rates for non-infectious incidents. Future coordination between government agencies and universities may aid state and local health departments as they develop systems for reducing infectious disease and environmental health hazards [19,20]. Overall, our findings raise the following question: Could incident reporting systems, similar to the one used in Ohio childcare settings, be leveraged to better protect children, childcare staff, and workers in other industries [21] during this pandemic and the next?

## Figures and Tables

**Table 1:** New COVID-19 cases by week in Ohio childcare programs from 15 August 2020 to 1 January 2021.

	Statewide		Childcare Overall (Workers and Children)		Childcare Workers		Children in Childcare	
	Cases	Test Positivity Rate	Cases	% of Statewide Cases	Cases	% of Statewide Cases	Cases	% of Statewide Cases
Weeks 1–10: Statewide SARS-CoV-2 Test Positivity Rate ≤5%								
15–21 August 2020	6297	4%	50	0.79	40	0.64	10	0.16
22–28 August 2020	7993	4%	26	0.33	16	0.20	10	0.13
29 August–4 September 2020	7714	4%	31	0.40	26	0.34	5	0.06
5–11 September 2020	6811	4%	32	0.47	25	0.37	7	0.10
12–18 September 2020	6178	3%	35	0.57	28	0.45	7	0.11
19–25 September 2020	6984	3%	44	0.63	26	0.37	18	0.26
26 September–22 October 2020	8687	3%	48	0.55	27	0.31	21	0.24
3–9 October 2020	11,454	4%	59	0.52	38	0.33	21	0.18
10–16 October 2020	14,316	5%	81	0.57	53	0.37	28	0.20
17–23 October 2020	18,315	5%	103	0.56	64	0.35	39	0.21
Weeks 11 to 20: Statewide SARS-CoV-2 Test Positivity Rate > 5%								
24–30 October 2020	23,997	7%	125	0.52	92	0.38	33	0.14
31 October–6 November 2020	39,872	9%	177	0.44	135	0.34	42	0.11
7–13 November 2020	55,903	12%	307	0.55	218	0.39	89	0.16
14–20 November 2020	61,378	13%	395	0.64	289	0.47	106	0.17
21–27 November 2020 ^a^	59,064	14%	261	0.44	193	0.33	68	0.12
28 November–4 December 2020	70,565	16%	374	0.53	281	0.40	93	0.13
5–11 December 2020	63,326	15%	404	0.64	285	0.45	119	0.19
12–18 December 2020	55,444	14%	330	0.60	229	0.41	101	0.18
19–25 December 2020 ^a^	42,896	13%	201	0.47	142	0.33	59	0.14
26 December 2020–1 January 2021 ^a^	51,236	14%	192	0.37	154	0.30	38	0.07

Notes: Childcare case data are from the Ohio Department of Job and Family Services Serious Incident/Injury Reporting System (compiled weekly). Overall COVID-19 case data are from the Ohio Department of Health COVID-19 Dashboard as of 19 January 2020. The weekly SARS-CoV-2 test positivity rates are the 7-day moving average from the last day of the time range. ^a^ Weeks include holiday closures of most childcare programs due to Thanksgiving, Christmas, and New Years. Therefore, reporting to the JFS Serious Incident Reporting System may not accurately reflect case rates in these weeks.

**Table 2:** Asymptomatic testing for SARS-CoV-2 with RT-PCR among childcare workers in 46 childcare programs in Ohio, September–December 2020.

Testing Month (2020)	Total Tested	% Positive ^a^	Positive	Negative	Indeterminant or Ineligible
September	24	0.0%	0	24	0
October	118	0.0%	0	110	8
November	133	1.57%	2	125	6
December	83	1.25%	1	79	3
Total	358	0.88%	3	338	17

Notes: All tests were conducted using FDA emergency use authorized kits through self-administered nasal collection methods. Indeterminant results were due to user error such as poor sample quality or incomplete registration of testing kit, and 9 of 17 individuals with indeterminant test results were successfully retested. Ninety-three individuals with confirmed results were retested according to study procedures. ^a^ Percent positive excludes indeterminant results (i.e., percent positive among total with confirmed results).

**Table 3:** Ohio childcare administrator concerns about COVID-19 from focus groups in Fall 2020.

1. Overall effort and financial costs associated with mitigation were high and unsustainable	
Representative quotes	“I think the lower ratios help, but not everyone can do that. You can’t financially do it. There’s no way you can financially do it unless you have other funding sources.” “The funding is definitely key because I mean, I know our staff feels safer that the lower ratios that we’ve stayed at and I feel like the longer that we can keep those lower ratios, the safer we all feel and stay and the easier it is for the kids to even do the social distancing through the day so that the funding obviously is key there because there’s only so many spaces you can put kids.” “Oh, I think for us is that the amount of kids that we have enrolled, we went from….our center can hold up to 92, 93, 94 kids. Now we only have 40 and then still trying to keep all of our staff on board and make sure they’re able to come to work and be able to, you know, take care of their household by having a job with us” “My fear is when the grant money runs out. And if I still don’t have staff and I’m forced to bring in more kids, does that burn my staff out more? ….right now, I am not in a [financial] loss…thankfully because of the pandemic support payments.”
2. It was hard to maintain a reliable supply of competent staff	
Representative quotes	“I mean, the difficulty is keeping things staffed and keeping groups separate for us right now. That’s the biggest challenge we have.” “We were having trouble finding teachers that are qualified for [name of accrediting agency]. So, we’ve gone through how many teachers in the last two months? 13. Some of them only were there a week” “ …we are struggling with hiring staff and, you know, as we’re trying to get back to allowing more children to come in …” “I have zero subs. If somebody gets sick, I actually told the parents and put it in my handbook that their class is gonna be canceled. There’s nothing I can do. My hands are tied.” “…it’s really been difficult for us to find good quality staff and teachers. And I tried calling the colleges around in the area…trying to get a list of people that want to work, along with all the job boards. And I still can’t get anybody to come in for an interview….it creates a lot of stress, the difficulty in hiring staff especially during the pandemic.”
3. Supply chains for PPE, cleaning supplies, and COVID-19 testing were challenging	
Representative quotes	“Yeah, I mean, everybody is saying the same thing that we’re finding out, too. Like we used to buy a box, a case of gloves, you know, for $26. Now it costs $83. And I heard they’re going up sometimes to $130 and almost $200. But I’ve been buying since March 27th. So, I always buy double what we need so that we have it when we need it. Because you go to the store and sometimes you can’t find them. They’re not available. You can’t buy gloves, if you can’t buy gloves in order to change diapers, how do you do your job? The teachers can’t do it. There’s no hand towels. There’s no rolls of paper. There’s not none of those things. If the state said…’we’ll treat you guys like the hospitals because you’re providing service for the first responders, here’s a supply chain that you could go to.’ But that wasn’t available.” “I think just being able to get the sanitizing things that we need. There is, you know, a shortage of wipes and sanitizing spray and all of that to the point to…we had to make our own here at the [name of child care program]. And it’s still an issue because you still can’t find this stuff, and even if you want to make your own, you still can’t find the alcohol or, you know, whatever you need.” “It’s just so hard to find stuff so we have been doing the bleacher water solution because we haven’t been able to find Lysol wipes and things like that.” “…The Lysol wipes, diaper gloves. Those I have paid… oh my gosh… like three times what I paid before COVID….” “My director and I kind of, you know, go to stores every other week, just try it, hoping it’s [disinfectant wipes] there and hoping that, you know, we can go through the checkout line two or three times to get enough.” “It is very hard. You know, I go first thing in the morning, sometimes down to [name of chain superstore], you know, to look for these supplies.”

## Data Availability

Passive surveillance data (from state agencies) are presented in the tables. De-identified individual data from active surveillance (RT-PCR diagnostic tests) and focus group conversations will not be made available.
